# From emotional signals to symbols

**DOI:** 10.3389/fpsyg.2024.1135288

**Published:** 2024-04-02

**Authors:** Ulrike Griebel, D. Kimbrough Oller

**Affiliations:** ^1^School of Communication Sciences and Disorders, The University of Memphis, Memphis, TN, United States; ^2^The Institute for Intelligent Systems, University of Memphis, Memphis, TN, United States; ^3^The Konrad Lorenz Institute for Evolution and Cognition Research, Klosterneuburg, Austria

**Keywords:** origin of language, evolution of language, development of language, infant vocalization, emotional expression

## Abstract

The quest for the origins of language is a diverse enterprise, where research from a variety of disciplines brings area-specific ideas and area-specific terminology to bear. This variety often results in misunderstandings and misconceptions about communication in various species. In the present paper, we argue for focus on emotional systems as the primary motivators for social signals in animals in general. This focus can help resolve discrepancies of interpretation among different areas of inquiry and can illuminate distinctions among different social signals as well as their phylogenetic origins in animals and especially in humans. We advocate, following Jaak Panksepp, a view wherein the Seeking System, the endogenous tendency to search and explore, is the most fundamental emotional motivation. The Seeking System forms the basis for flexible, voluntary, and exploratory control of motor systems and makes much of learning possible. The relative lack of vocal learning and expression in nonhuman primates contrasted with extensive vocal learning and expression in humans began, we propose, with the evolution in ancient hominins of a necessary foundation for the many subsequent capabilities required for language. That foundation was, according to the reasoning, naturally selected in the form of neurological connections between the Seeking System and mechanisms of glottal/phonatory control. The new connections allowed ancient hominins to develop flexible, endogenous vocal fitness signals produced at very high rates and including large numbers of discrete syllables, recombinable to form syllable combinations with many prosodic variations. The increasing sociality of hominins supported evolution of massive expansion in the utilization of these flexible vocal forms to allow development of words and sentences.

## Introduction

### Emotions as functional motivational states: the seeking system as a foundation

Emotions are assumed by many to apply only to humans or only to animals that have highly developed brains. We advocate a view introduced by Jaak Panksepp, where at least some emotions as well as other affective states, such as hunger or the sensation of cold, are thought to apply at least to most vertebrates (Panksepp, 1998). Others have sought recently to characterize biochemical and genetic *foundations* of emotions in invertebrates such as the fruit fly (Anderson and Adolphs, 2014; Gibson et al., 2015; Anderson, 2022). Even in unicellular animals such foundations have been proposed to include conserved biochemical processes that generate exploratory foraging activity (Hills, 2006). Panksepp hypothesized that such primitive, apparently endogenous actions formed a basis for the evolution of more elaborate emotions in mammals and birds, implemented in specific neurotransmitters and largely subcortical neural circuitry.

In the present paper, we focus mostly on communication in humans and nonhuman primates (NHP), applying Panksepp’s theory of affect as a basis for understanding primate vocalizations as expressions of emotion. But in other species, especially birds, calls and songs also provide evidence of emotional expression (Marler and Evans, 1996; Papini et al., 2018). For those who may object to the use of the term emotion in this way, it is possible to substitute “motivation” or “affect” for emotion. The focus is on internal states that underly certain behaviors. Our paper proposes that Panksepp’s theory may provide a critical missing piece in the attempt to explain how ancient hominins broke away from the primate communication background through evolution of a powerful motivation to vocalize exploratorily, thus forming a foundation for the evolution of vocal symbology and thus, for language.

In this view, emotions and other affective states orchestrate all kinds of behavior. Emotions stimulate animals to do what is advantageous, reflecting action strategies honed over eons (Damasio, 1999). For example, when encountering danger, emotions such as rage or fear prepare mammals to take measures of attack, retreat, or freezing. Emotions also motivate mammals to reveal their emotional states vocally, providing information about what they may be likely to do, a fact that has been recognized since Darwin’s characterization of emotional expression (Darwin, 1872).

Prior publications have also pointed to emotional foundations for communication and for language (Turner, 1996; Jablonka et al., 2012). These works have even emphasized “special” emotions of humans that appear to have emerged as a consequence of the extreme sociality of human groups. Panksepp also has acknowledged special human emotions that emerge from interactions of the basic emotions with cognitive capabilities and cultural adaptations that seem unique to humans.

Our paper focuses primarily on vocalization, because it is vocal language we wish to account for in evolution and development. This focus is not intended to discount the multimodality of communication in humans and other animals. Communication in primates includes facial expressions, body postures, and gestures as well as many intensity cues that modulate interpretation, which is also heavily modulated by the perceived context of communicative actions (Iverson, 2010; Taglialatela et al., 2011; Fröhlich et al., 2019). Emotions and other affective states, in accord with our proposal, are at the root of communication in all its modalities. It is also important to recognize that a dichotomous view of gesture as intentional and vocalization as emotional in NHP is not justifiable (Heesen et al., 2022b). The flexibility of usage of both gesture and vocalization in the great apes makes clear that their emotional nature does not negate their intentional and flexible usage, topics that have been richly explored in recent research (Scheider et al., 2016; Liebal and Oña, 2018; Oña et al., 2019; Heesen et al., 2022a). Research has also reported that the great apes can vocalize intentionally based on what is known by the receiver and that there is considerable contextual flexibility in how and when vocal signals are given (Crockford and Boesch, 2003; Schel et al., 2013; Clay et al., 2015; Crockford et al., 2018).

Panksepp (1998, 2005) viewed affective states, including emotions, as intrinsic motivations rather than merely as action patterns in response to environmental events. Emotions motivate flexible, targeted action in diverse external conditions, often triggered by subtle internal or external cues and often lasting over considerable periods, during which emotional states may remain activated even though the arousing event has passed. Consider how fear can last for many minutes after being triggered by an odd sound occurring in an unfamiliar place. The persistence of affective states long after the stimulus that instigates them is demonstrable in humans (Heller et al., 2015; Ivanov et al., 2020) and even in the fruit fly (Anderson, 2022).

This paper is an exploration of how emotions and other affective states such as hunger or pain motivate vocal communication, and further, how and why human vocal communication has properties that are in some ways held in common with other primates but in other ways contrast dramatically with communication in any other species. We share much of this viewpoint with other investigators (Owings and Morton, 1998; Owren et al., 2011). In some cases the connections between particular emotional states and corresponding vocal acts seem straightforward, as in aggressive sounds that can occur before or during physical conflicts. But natural selection has produced ways that animal vocal signals can be adjusted flexibly for context. Recent research has overturned previous assumptions that primate vocalization was limited to inflexible signals of emotions. For example, chimpanzees produce alarm calls in ways that are tuned to the awareness of others (Crockford et al., 2012) and are more likely to announce the presence of food if a friend is arriving than if some other individual is arriving (Schel et al., 2013). Other animals, for example, chickens have also been shown to modify alarm calls based on whether other chickens are nearby (Evans et al., 1993; Evans and Evans, 1999).

We propose that human vocalization has been naturally selected to be connected with an emotion that is typically not even treated *as* an emotion, the “Seeking System.” Panksepp and his colleagues proposed that the Seeking System is the “granddaddy” of the emotions (Panksepp and Biven, 2012), motivating organisms, even in the absence of immediate conditions requiring action, to search and explore their environments. Seeking sometimes resembles foraging in unicellular animals (Hills, 2006), but things are more diverse in multicellular animals such as mammals, where search and exploration can produce not only a wide variety of experiences potentially beneficial to survival and reproduction but also a sense of pleasure to keep animals seeking even when they do not seem to need to, a point that is illustrated as fundamental in the research of Panksepp and his colleagues (Panksepp et al., 1984; Ikemoto and Panksepp, 1999; Panksepp, 2005, 2011; Burgdorf and Panksepp, 2006).

Panksepp and others have shown that laboratory rats, for example, repeatedly explore every nook and cranny of an empty box (Olds and Milner, 1954; Panksepp, 1998). They seem to enjoy this exploration, and the activity generates neuro-chemical pleasure signatures (especially dopamine). Rats with an implanted electrode in a Seeking System tract, the medial forebrain bundle of the lateral hypothalamic area, have been found to repeatedly self-stimulate by a lever press and explore until they are exhausted. Seeking System activity is described as including “…intense and enthusiastic exploration and appetitive anticipatory excitement …” (Panksepp, 2010, p. 537).

Mammals show a deep motivation to understand the world, to watch, touch, listen, taste and smell, being motivated by curiosity and interest, a condition that regularly occurs while they are awake, looking for novelty as illustrated in the research of Panksepp and colleagues (e.g., Panksepp and Biven, 2012). Seeking can also be thought of as driving planning, strategizing, and imagining. Humans appear to seek information through travel and reading, and the search for entertainment can also be viewed as motivated by the Seeking System, although we know of no experimental work with humans to illustrate this apparent tendency. The neurological and neuro-chemical foundations of the Seeking System were described in a variety of animals through extensive research reviewed by Panksepp (1998, 2005, 2011) and Panksepp and Biven (2012).

The Seeking System is not present in traditional emotion categorization schemes, perhaps because behavioral research has often been limited to consideration of “stimulus and response.” The endogenous, active organism in Panksepp’s (1998) theory constitutes a dramatic departure from strict behaviorism, because so much of what animals do is *not* seen in his emotion-centric conception as reactive to the external environment, but as the internally-generated behavior of an organism motivated to explore. Panksepp (1998) and Panksepp and Biven (2012) acknowledged repeatedly that behavior is responsive to the external environment, but they argued that animals seek information regardless of external circumstances. They referred to the work of Olds and Milner (1954), who had concluded that electrical stimulation of the septal area “is rewarding in the sense that the experimental animal will stimulate itself in these places frequently and regularly for long periods of time if permitted to do so (p. 419).”

We contend that vocal communication in most primates is predominantly motivated by emotions that are tied to *immediate needs* and triggering events. This interpretation is consistent with the original Darwinian description of emotional expression (Darwin, 1872). It does not appear that NHP *vocalizations* generally require involvement of the Seeking System, although the Seeking System does, in accord with Panksepp’s (1998) theory, motivate NHP to develop intelligent, forward-looking strategies for foraging, hunting cooperatively, warfare with neighboring troops, alliance formation to overthrow tyrannical alphas, and so on (de Waal, 2016, 2019). Vocal communication instead appears to be motivated in NHP by emotions of the here and now, such as fear or rage and others to be considered below in fuller discussion of Panksepp’s theory.

Human vocalizations are sometimes also motivated by immediate emotional triggering events, for example, by fear or rage, but the great majority of adult human vocalizations do not require such triggering. Human vocalizations regularly merely require, in accord with Panksepp’s model, motivation through the Seeking System. We propose the Seeking System motivates human infants in the first months of life to produce ~3,500 speech-like vocalizations or “protophones” per day (Oller et al., 2019, 2021), the vast majority of which are directed to nobody (Long et al., 2020), but seem to constitute endogenous exploration of the vocal capacity (Oller and Griebel, 2021). We propose further that the connection of vocal control to the Seeking System in hominins established first in ancient hominin infants.

In accord with Panksepp’s theory, we propose that because of the connection of the Seeking System to human vocalization, we can explore vocalization and at other times vocally express any emotional state flexibly with those same sounds, and in maturity, with an indefinitely large set of words and sentences. The Seeking System, in our interpretation, allows human vocalization to require no targeting of immediate benefits, and thus frees the human vocal capacity for expressions that can bear any social function or “illocutionary force” (Austin, 1962; Oller, 2000), a concept elaborated below. Ultimately human vocal expressions become symbols that can refer not only to entities in the here and now, but to entities in the past, the future, or the imagination. Our proposal is that the connection of human vocalization to the Seeking System makes these advances beyond NHP communication possible.

### How is language possible, and indeed how is nonhuman animal communication possible?

Biologists have often wondered how communication signals could have evolved at all, since so much of communication appears to be about conflict, competition, and deception (Zahavi, 1975; Krebs and Dawkins, 1978; Knight, 2016). Wouldn’t signals deteriorate before they could stabilize because of conflicting interests? Maynard Smith and Harper (2003) argued that signals stabilize because in general they benefit both the sender and the receiver, conveying useful information about the sender’s affective state. If a baby communicates distress because of hunger or fear, it is in the interest of the mother to react by giving care, since the promotion of her genes is at stake. The benefits of this type of communication can be extended to relatives in general (Hamilton, 1963) as well as to unrelated group members who might return the favor later in a tit-for-tat arrangement (Trivers, 1971).

One might expect aggressive signaling to give the sender the edge over the receiver, but aggressive signaling is also important for the receiver, who can benefit from knowing how motivated the sender is. The receiver might wisely choose to back off after perceiving a highly motivated aggressor. Protection of home often wins over threatened attack because the home protector may have more to lose and have higher motivation to fight (Hoefler, 2002). In general it is to the advantage of both parties in aggressive exchanges to display their motivation regarding a possible fight and to assess the motivation of the opponent.

Much research has addressed cheating and manipulation in such communicative acts as reviewed by Oller and Griebel (2021, this Frontiers Topic). We propose that communication systems are evolutionarily stable in part because animals display and assess each other’s affective states and benefit from the interchanges in guiding their actions. The existence of deception does not contradict this view. In stable communication systems, deception has to be rare, because otherwise signals would disintegrate—they have to be generally reliable indicators of emotional states and the related intentions. Experience in social groups can also play a major role in maintaining stability of shared signals. Group members tend to learn whose emotional signals and communicative intentions to take seriously. For example, inexperienced youngsters’ alarm calls may be ignored (Cheney and Seyfarth, 1988). Thus, vocal signals must be recognized by animal receivers as revealing affective states, but their responses can be intelligent and flexible based on what they know about the sender, the sender’s perceived intentions, or the situation.

NHP vocal signaling appears generally to elicit responses presumably consistent with innately determined purposes of the sounds, such as maternal caregiving in response to infant distress calls. The same is true in the human case during early infancy, but across development the producer’s intended function and the listener’s interpretation of crying can change because human infants learn to cry manipulatively rather than only as an expression of distress (Green et al., 2011; Chóliz et al., 2012). By human adulthood, crying and laughter can even be expressed in ways that are contrary to their innate purposes. Think of derisive laughter or crying in relief. We propose that this flexibility and the seemingly unlimited emotional flexibility of linguistic expression are possible in humans because all vocalizations in humans can be motivated through the Seeking System, which allows vocal flexibility to an extraordinary degree.

Understanding the relation between the Seeking System and human vocalization may provide a key to clarifying both similarities and differences between human and NHP vocalization. If we focus on the Seeking System, we may be able to sort out many prior difficulties of interpretation that have led animal communication research down unproductive paths, directing attention away from fundamental differences between vocal communication in humans and nonhuman animals, while also failing to address the most salient similarities. We propose that the relations between vocal communication in humans and nonhuman animals need to be restructured in both conceptual foundations and terminology, taking account of a role for the Seeking System. The suggestion that the Seeking System may have come to be connected to a far greater extent with vocalization in humans than in NHP is consistent with the fact that humans have extensive neural connections between laryngeal motor cortex (LMC, located in area 4 of the primary motor cortex) and laryngeal motoneurons, while NHP have been shown to possess little if any such connection (Jürgens, 1995; Kumar et al., 2016; Simonyan et al., 2016). Laryngeal motor neurons in monkeys appear to have indirect connections from area 6 of the premotor area, but their destruction does not appear to impact species-specific calls (Simonyan et al., 2016). Consequently it seems possible that the Seeking System in humans (perhaps from its lateral hypothalamic site) may be shown to have strong connections with LMC.

## Explaining discrepancies in the comparative literature on vocal communication

### Interpretive and terminological misunderstandings about signaling repertoires

One vexing issue in description of signaling systems is that the numbers of communication signals in species repertoires have been reported to be vastly different. For example, in a broad review of primate communication literature, chimpanzees were reported to possess from 7 to 27 different vocal types across studies, and similar discrepancies were found for other primate species (Sutton, 1979). Obviously, the criteria for counting differed across the studies reviewed.

Perhaps the main reason for such discrepancies is that signals along a particular dimension of emotional expression grade from low to high intensity, and they can show regime shifts of sound quality along each such dimension. Squirrel monkey vocalizations, for example, have been reported to pertain to five functional dimensions (protest, challenge, social contact, group action, and alarm), with gradations on each one corresponding to apparent regime shifts, yielding the misleading impression of many more than five distinct functional categories (Jürgens and Ploog, 1976; Ploog, 1992). Interestingly, at very high intensity, all the emotional dimensions of squirrel monkeys tended to collapse acoustically, yielding a single loud, dysphonated sound. At very low intensity, all the dimensions seemed to collapse to a single very quiet sound, so that middle range intensities yielded best discriminability (Jürgens, personal communication; and see Oller, 2000, pp. 339–355).

Especially in very social animals such as primates, it is important for receivers to assess the intensity of the emotions driving signals. This intensity is reflected in graded vocalizations, with seemingly categorical shifts, especially with sudden changes from periodic phonation at low intensity to noisy dysphonation at higher intensity (Owren and Linker, 1995; Owren and Rendall, 2001; Rendall and Owren, 2013). The sender’s degree of motivation can also be reflected in the number of repetitions of vocalizations.

Failure to take emotional motivation into account appears to have contributed to the narrative in much animal communication literature portraying animal vocalizations with linguistic terms. The foundational research on this topic was based on observations of vervet monkeys, *Chlorocebus pygerythrus* (Seyfarth et al., 1980). The research inspired the widespread claim that various animals have “semantic” vocalizations in the form of predator-specific alarm calls (Seyfarth et al., 1980). The term was misleading, as will be seen below, having been largely rejected by more recent investigators (Owren et al., 2010; Rendall, 2021) and having been substantially weakened in more recent time even by the originators of the claim (Cheney and Seyfarth, 1996). Even so, additional researchers have interpreted distributional differences in the acoustic characteristics of alarm calls of a variety of species in the presence of different predators as indicating “referentiality,” “functional referentiality” or “representation” (Gozoules et al., 1995; Hollén and Manser, 2007; Furrer and Manser, 2009; Rogers et al., 2018, and see a variety of papers in a volume by Stegmann, 2013).

Yet the term “predator-specific alarm calls” of vervets, which lay at the basis of the semanticity claim, was misleading all along, since it has been widely acknowledged that the relevant calls are not specific to predators. They are also commonly used in intra-specific aggression, that is, the same calls occur during fighting and threats by one vervet in conflict with another (Price et al., 2015). This fact was even noted briefly in the article that first presented the claim about semantics in vervet monkey calls (Seyfarth et al., 1980). The confusions about alarm calls are placed in historical perspective in two additional articles in this Frontiers Topic (Locke, 2021; Rendall, 2021).

The tendency for calls associated with predator danger to segregate into acoustically different groupings can easily be interpreted in a way that requires no appeal to semantics. Instead, the segregation can be interpreted as relying on emotional expressions occurring differentially because of the different intensities of fear or anger that can occur at typical sighting of particular predators (Oller and Griebel, 2014). Different degrees of fear and anger can mix in ways that are appropriate for particular situations and may yield different sounds in response to a predator that may typically be seen crouching on the ground as opposed to one that may be soaring in the air or slithering in the grass. The extent of the alarm-caller’s emotional reaction may segregate probabilistically so that one type of predator tends to cause more alarm than another simply because they may tend to be nearer when detected or may tend to be approaching faster when detected. Given the existence of regime shifts in graded vocalizations, it seems possible that typical alarm calls to one type of predator might misleadingly seem categorically different from those to another, only owing to probabilistic differences in the typical intensity of the reaction.

It has not even been proven that the producer of a so-called alarm call *intends* the vocalization to constitute an alarm. A human observer may think of the sound as an alarm, and conspecifics of the caller may respond *with* alarm, but we know of no evidence indicating that the animal produces an alarm call based on anything other than fear and/or anger. An increased tendency to produce alarm calls when kin are nearby could be interpreted as simply indicating the sender feels more fear or anger knowing their kin might be in danger. Of course it is an empirical question what is in the mind of an animal signaler (long-term plans and thoughts could indeed be involved), but an alarm call itself does not include semantic information about the mind of the signaler. For the receiver, it only supplies emotional/illocutionary information, reflecting the caller’s state and immediate intentions. Other concerns about interpretation of alarm calls were expressed by Kaplan (2008) based particularly on evidence from the Australian magpie. Her conclusion was that the various calls that have been termed “referential” appear to be generated principally in the midbrain, offering little support for any interpretation of complex cognition being involved. Still, while an animal signal itself may reflect only the type and intensity of the sender’s emotion/illocution, the listener may bring to bear contextual information and prior experience in determining how to react.

Different distributions of animal food calls in the presence of different edibles have also been interpreted as referential or functionally referential (Evans, 1997; Evans and Evans, 1999; Rogers et al., 2018), but in our opinion this interpretation is subject to the same concerns as the interpretation of animal alarm calls. The empirical evidence does not appear to prove that the calls themselves contain reference. As in the case of alarm calls, the possibility remains that the differences among the calls in the presence of different kinds of food are the products of different emotional reactions of the vocalizers to the different food types. This does not mean, however, that the food calls have no flexibility since, for example, their production has been shown in some instances to reflect audience effects (Hauser and Marler, 1993; Evans and Evans, 1999; Schel et al., 2013). Nor does the possible lack of reference contained within the calls themselves rule out conspecific listeners’ reactions being differentiated based on possible learning by the community of listeners about the likelihood that different foods may have stimulated the senders’ differentiated vocal reactions.

Our conclusion is that neither food calls nor alarm calls in animals have thus far offered evidence of semantics. For any vocal act to be semantic it is required that it be motivated by a wide variety of states and intentions, for example to involve simple naming in the absence of alarm or in the absence of any kind of food and in the absence of any particular emotional state. Human linguistic reference is *never* *limited to* specific circumstances of physical context or emotional state. The failure so far to produce convincing evidence of semanticity in animal communication in the wild does not appear to be a failure of methodology or of investigator persistence. It appears instead to be a failure due to inappropriate goal-setting—researchers have sought to show advanced features of human language (especially semanticity) in wild animals without first taking stock of the fundamental principles of cognition and behavior that are required by such language features. The same researchers have largely left aside the investigation of fundamental *differences* between human and nonhuman animal communication.

There are, however, ways to compare human and nonhuman animal communication profitably. To find both similarities and differences between them, we can look to non-linguistic human communication modes that have much in common with nonhuman animal vocalization, such as the human non-verbal vocal repertoire, facial expressions, and non-symbolic gestures that are clearly associated with emotions or other affective conditions. Human infant crying (which continues intermittently in maturity in modified and much more flexible form), for example, has much in common with the calls of other mammals, since crying expresses distress caused by pain, fear, or isolation (Owings and Zeifman, 2004). Similarly, laughter has been interpreted as occurring across many primates, and although the acoustic form of laughter differs across species, it seems clear there is homology involved (Davila Ross et al., 2010). As with crying or screaming, the cross-species similarity in laughter is grounded emotionally—laughter’s central function is always the emotional expression of social connectedness or joy, which in NHP tends to occur in response to tickling or rough and tumble play (Panksepp, 2000).

Laughter and crying have sometimes been referred to as “fixed signals” (Lorenz, 1951; Tinbergen, 1951), but this is an overly restrictive term, because these sounds can show substantial gradations of intensity. Laughter and crying are indeed relatively fixed in that they each have a limited range of variability regarding their functions, either the expression of a positive social emotion or the expression of distress. In this way human and nonhuman animal vocalizations have something fundamental in common.

In contrast, even the precursors to language seen in human infancy are not constrained to expression of particular functions the way crying and laughter are. On the contrary, from the first months of life, human infants express the full range of affective valences from positive to neutral to negative with each of the phonatory vocal types (e.g., squeals, vowel-like sounds, growls) known to be precursors of speech (Oller et al., 2013; Jhang and Oller, 2017). Infants can shift in just a few moments between emotional states, and they can use any one of their vocal types to express any emotional valence. Thus one may observe an infant to produce a squeal with a smiling, happy face, then later a squeal with a neutral face, suggesting neutral emotional valence, and later yet with an obvious frown indicating discomfort or annoyance. Human observers make consistent judgments about the differing emotional states accompanying the very same vocal types on different occasions (Oller et al., 2013). All elements of mature human languages require this kind of functional/illocutionary flexibility—every syllable, word or sentence must be possible to produce in any emotional state. In fact, each element of language must be possible to produce merely based on interest in the sound itself. Infant protophones produced this way are judged consistently by human observers to constitute exploration (Long et al., 2020; Oller et al., 2021), and we propose this exploration depends on connection to the Seeking System.

The endogenous nature of human infant vocal exploration is indicated partly by the facts that (1) the great majority of protophones are directed to nobody (Long et al., 2020), (2) they express a variety of emotional states (Stark, 1981; Shimada, 2012), (3) much of the infant vocalization occurs when infants are alone in a room (Delack and Fowlow, 1978; Oller et al., 2019), and (4) even profoundly deaf infants produce massive numbers of protophones (Iyer and Oller, 2008). Most protophones seem to constitute a kind of vocal play (Stark, 1980), a fact that is supported by observations from human coders of hundreds of randomly-sampled segments from all-day recordings across the first year of life as well as from extensive longitudinal research with laboratory-based audio-video samples (Oller et al., 2021; Long et al., 2022). Similarly, adult humans produce speech commonly (often muttering to themselves) for no immediate social purpose, perhaps as a sort of anticipatory practice, expressing interest in both the speech itself and the semantic content it could at some point transmit to an imagined listener. Yet even recent efforts to explore the possibility of vocal functional flexibility in nonhuman animals (Clay et al., 2015; Dezecache et al., 2020; Taylor et al., 2022, 2023) have not reported nonhuman apes producing any vocalization exploratorily. So far neither vocal exploration nor semantic communication has been found in NHP vocal communication, and it would be more fruitful to focus on similarities and differences between signal types that, like crying and laughter, are in general based on similarly constrained functions.

The inappropriate attempt to shoehorn the vocal communications of nonhuman animals into categories of human language is exacerbated by failure in much animal communication research to draw the critical distinction between situational context and function of communication signals. Some research has considered *only* situational context in categorizing signals, ignoring social functions entirely. For example, the term “food calls” or “food associated calls” (Hauser and Marler, 1993; Clay and Zuberbühler, 2009) suggests that calls produced near food or in anticipation of eating are *about* food. It is much more plausible that they may be expressions of positive excitement or of some other emotional state that can be expressed in the absence of food. Other research by some of the same authors makes clear that vocalization in the presence of food by mammals and birds does not provide referential information (Clay et al., 2012).

The same behavioristic approach can lead to aggressive or fear expressions being categorized as predator-specific alarm calls, because they *can* occur in the context of sighting a predator, although they also occur in intra-specific aggression. NHP signals can be used in a variety of different contexts, because communicative functions, such as aggression, appeasement, courtship, play, and fear, are transmitted in contexts as different as resting, traveling, and feeding. Categorizing signals by situational context alone, instead of also addressing social function, creates confusion in comparative communicative research. Social functions/illocutions of the vast majority of NHP vocalizations can be best explained as being driven by emotions that were naturally selected to serve particular social functions in the immediate present. It has not been proven that NHP vocalizations ever express any kind of semantic content, which requires by definition flexible control and learning of conventional symbols.

### Roots of confusion about language and nonhuman animal communication in radical behaviorism

The confusions associated with trying to categorize animal vocal communications in terms of human language categories have also been exacerbated by a longstanding tendency in the study of animal behavior to focus on *and only on* observable, countable events, and to ignore explicitly the internal states of animals and motivations that drive behavior (Watson, 1913; Skinner, 1957). Situational context has been at center stage in radical behaviorism. Internal states of organisms have been treated as irrelevant. But there is no accounting for how and why behavior occurs without addressing the states within animals that are the immediate and necessary drivers of behavior. Situational context can help us to *infer* internal states, but to explicitly ignore internal states is to abrogate the ultimate responsibility of ethological science (Lorenz, 1971), which is to explain behavior and its evolution.

One of the results of the tendency to confine behavioral science to observables is that scientists have often seemed to take deeply contradictory stands on whether animals possess emotions at all (see commentary in Panksepp, 2005; Panksepp and Biven, 2012). Biologists who have actually wanted to study emotions during the century of the behaviorist paradigm have sometimes complained that animals are treated as simple stimulus–response machines without any emotions or minds, while psychologists trying to explain human behavior may complain that humans struggle with their emotional heritage, their lowly “animal” side, which is thought to run afoul of the humans’ highly evolved rational cognitive abilities. We tend to agree with Panksepp (1998) that there can be no cognition without affect, that there can be no learning without emotional motivation, and that emotions are modulated by learning and memory in both humans and many other animals. In this view, emotion and adaptation by learning had to evolve together, like two sides of a coin.

While visual and chemical signals are also used to communicate emotional states, the vocal mode is naturally connected to the breathing apparatus in vertebrates, and consequently to arousal, which can reflect levels of emotions through respiratory pressures, volumes and rates. Phonatory mechanisms rely on respiration as the force to drive vibratory patterns of larynx, syrinx, pharyngeal pouches, air sacs, etc. (Conrad and Schönle, 1979; Kent, 1998; Suthers, 1999; Farmer, 2006; Schusterman, 2008). Vocalization can thus occur to some extent accidentally in circumstances of high arousal and intense emotion. Consequently we surmise that vocalizations, being naturally associated with affective states through breathing, have been particularly sensitive to selection pressures that could have differentiated vocalizations to express individual affective types and for elaborating them into graded signals.

Visual systems of communication can be flexible and can include relatively large repertoires, as in the case of the chromatophore system in cephalopods (Messenger, 2001; Byrne et al., 2003) or gestural communication in NHP (Call and Tomasello, 2007; Call, 2008). But with the exception of human sign languages (Stokoe, 1960), only vocal sound production has produced massively complex categorical signal types, and these have occurred only in human languages and in certain species of birds and marine mammals showing elaborate songs with an amazing variety of “syllable” types (Helweg et al., 1992; Marler and Slabbekorn, 2004).

## Emotions at center stage

### Panksepp’s view of emotions

While widely recognized models of emotional systems (Ekman, 1994; LeDoux, 1998; Damasio, 1999; de Waal, 2019) show substantial overlap with Panksepp’s (1998) model, they also differ importantly. LeDoux (1998), for example, has resisted even acknowledging that emotions exist in nonhuman animals, and in general other models do not provide a basis for characterizing the origins of vocal emotional expressions in either humans or nonhuman animals. Panksepp pioneered characterization of emotions and other affective states (including hunger, thirst, pain) both theoretically and in neurobiological research.

While all of the other basic emotions of Panksepp’s model to be considered below are commonly recognized in some form, the Seeking System is an essentially new concept. There has been mention of an exploratory “drive” that causes animals to investigate their environments, but the description of this “drive” prior to Panksepp was vague and largely undefined neurologically (Montgomery, 1954; Glanzer, 1958). Recent modern computational modeling research includes much interest in curiosity and exploratory behavior (e.g., Oudeyer and Smith, 2016), but Panksepp (1998) and Panksepp and Biven (2012) have supplied the most extensive characterization of the Seeking System.

Panksepp described the Seeking System as the most fundamental emotional system, motivating all animals to explore. The Seeking System presumably also plays a crucial role in innovative behaviors such as tool use, and we have proposed specifically that it has been crucial in the evolution of symbolic communication/language, since exploration of vocalization is one of the fundamental foundations for language. Without it, other prerequisites of language could not have emerged. The argument about additional stages of evolution and development that require the foundational stage of vocal exploratory flexibility can be found in our previous writings (Oller, 2000; Oller et al., 2016), but for the present, we simply note that the following list of four capacities appear in human infants in the following order across the first year:

(1) exploratory production of vocal types that are not part of the innate repertoire (e.g., cry or laughter) along with production of those vocal types in any condition of affect; vocal types occurring in exploration, presumably motivated by the Seeking System, can be said to be decoupled from any of the traditionally recognized emotions;
(2) flexible, sustained, affectively positive vocal interaction with others, forming a foundation for understanding others’ affective states and minds;
(3) vocal imitative learning of new sounds not in the innate or exploratorily developed vocal repertoire; and
(4) associative learning of the production of acquired sounds in association with arbitrary circumstances or entities, i.e., primitive word learning.

All four of these prerequisites to symbolic word learning (Sinha, 2004) regularly appear developmentally in the order given in humans, where #2 depends on #1, #3 and on #2, and #4 on #3. There are a number of additional steps specified in the more elaborate version of our model (Oller et al., 2016). Our key contention is that when the Seeking System was naturally selected to be connected to the human vocal system, and thus exploratory vocalization began in ancient hominins, a door was opened to evolution of many subsequent language-necessary capacities. Similarly, modern human development begins with exploratory vocalization from the first day of life, driven, according to our proposal, by the Seeking System.

Panksepp has described the neural circuitry and neurotransmitters active in seven basic emotions (Panksepp, 1998, 2005, 2011). All are portrayed as largely subcortical in vertebrates, although all can be modulated by cortical influences and by interactions across the different emotions. It is important to keep in mind, though, that to date science has produced minimal knowledge about a large number of only recently recognized neurotransmitters found in the neuronal synapses (Grant, 2015; Zhu et al., 2018). While major types have been identified (oxytocin, serotonin, dopamine, estrogen, testosterone, and so on), we know very little about how they interact with each other and with all the other neurotransmitters about which virtually nothing is known. The picture is getting more complex as research shows that the effects of a single neurotransmitter can be diverse and highly time-and area specific (see, e.g., Young, 2012). Nevertheless, there is reason for optimism since neurophysiologists have begun to characterize emotion-like systems in model organisms such as fruit flies and mice, starting from behavior and continuing to neurotransmitters and genes involved in the expression of primitive emotional states (Tsien et al., 1996; Adolphs and Anderson, 2018; Jung et al., 2020). Panksepp postulated that together with sensory/perceptual input, seven basic emotions create a primary process consciousness in mammals, which can be elaborated into states of secondary and tertiary consciousness, at least in humans, by reflections about experiences and reflections about reflections (Panksepp and Biven, 2012).

### Summary of the seven emotions proposed by Panksepp

The first emotion in Panksepp’s (1998) theory may apply even to unicellular animals, but at least to multicellular animals:

1. Seeking: Panksepp described this addition to the traditionally recognized emotions as an affective state of exploration, a dopamine-driven seeking/expectancy/wanting system that energizes activities such as foraging or object exploration/play (not social play, which belongs to the Play emotion, below) and mediates anticipatory states. Seeking stimulates a positive reward system (demonstrated especially in rats, where his research was most extensive) different from rewards associated with other emotions. There are no specific vocalizations associated with seeking, but if the Seeking System is connected to the vocal capacity, as we propose it is in humans and in other vocal learning species such as some song birds and some marine mammals, then an indefinitely large range of sound types and gradations of each type can emerge as a result of seeking/exploration.

Three emotions are common to both social and non-social vertebrates and perhaps also invertebrates:

2. Rage: a state of anger often expressed vocally by growling, roaring, barking, or hissing, depending on the species.
3. Fear: a state of negative agitation, yielding responses of freezing or flight, often accompanied by whimpering or screaming.
4. Lust: a state of sexual interest, yielding mating behaviors, with vocal expressions sometimes including sighing, moaning, or other sounds suggesting positive arousal.

The third group of emotions is unique to social animals:

5. Panic/Grief: a state of sadness or terror based on isolation often leading to frantic search or, after extended periods, depression. A vocal expression is isolation calling, but after a longer period of isolation, vocalizations can include sobbing or wailing.
6. Care: a state inducing enjoyment in investing in the well-being of others, usually offspring, but also sexual partners, and allies. Behaviors include nurturing, cuddling, helping, teaching, and so on. Vocal expressions can be soothing sounds at relatively low intensity or celebration sounds of positive arousal.
7. Play: a state of pleasurable social connection in seemingly (i.e., momentarily) unproductive behaviors such as, tickling, chasing, and rough and tumble play, often accompanied by laughter.

These seven are the emotional affects. Bodily regulatory urges/states such as hunger, thirst, the need to defecate, and so on, are categorized as “homeostatic” affects in Panksepp (1998, 2010) work, and the pleasures and pains of externally provoked sensations such as sweetness, bitterness, heat, coldness, or physical injury, are treated as “sensory” affects. These non-emotional affective states can also yield vocal expressions, such as infant crying with hunger or pain or sighing in response to a pleasurable taste.

Panksepp (1998) described various inhibitory and excitatory interactions among the basic emotions. Behavioral evidence from mammals (including humans) in addition to introspection show that we can experience mixed emotions (de Waal, 2011; Larsen and McGraw, 2014; Hoemann et al., 2017). We can be torn between fight or flight, love or hate, and we often seek rational solutions to emotional conflicts, a state that Panksepp reasoned to often invoke the Seeking System in order to acquire information needed to create a balancing strategy for action. Seeking can recruit memories of prior experience acquired during prior seeking and can support informed strategic action in response to conflicting emotions. Panksepp also contended that the Seeking System can be recruited to serve the goals of other basic emotions such as Fear (seeking an escape route, a means of defense, etc.), Lust (seeking ways to impress a potential sexual partner), Rage/aggression (seeking ways to impress an opponent, to get the upper hand in a physical fight), and so on. Thus emotional states are not completely isolated from each other, although they all, according to his research, have isolable subcortical components and biochemical signatures in mammals. Interactions are obviously necessary since, for example, Fear has to interact with other emotional systems to produce reaction strategies. Everyone knows the feeling of being torn between fight or flight, and we recognize the fierce and seemingly fearless aggression of a mother with pups (in this case Fear is suppressed, presumably by Rage), the balance of Fear and Lust in courting, and the tabling of behaviors based on prior emotions in the face of imminent danger. In the case of danger, Fear may dominate every other emotion. Since vocalizations are motivated by affect/emotion, the occurrence of mixed emotions suggests that the apparent range and number of possible vocalizations may be increased substantially beyond that which would be expected if each vocalization type corresponded one-to-one to a single emotion/illocutionary type.

### Reward systems in communicative behavior

Just as adaptive behaviors like reproductive acts, parental care, social bonding, play, and exploration often produce pleasure, communicative acts also have a pleasurable component. They appear to release endorphins and boost our immune functions, e.g., via opioids (Benson, 2019). While pleasure is obvious in most cases of laughter during social play (Manninen et al., 2017) in humans and our primate relatives (Davila Ross et al., 2010) and in some other more distantly related species (Panksepp, 2000), it seems likely that courtship displays such as mating songs or territorial or social group choruses in, for example, canids and primates induce pleasure as well. It has been shown that singing in a choir releases endorphins in humans (Launay and Pearce, 2020).

Panksepp’s (1998) extensive review of evidence that seeking is pleasurable suggests exploratory vocalization could yield pleasurable sensations, just as exploration by smell or by touch in a variety of animals could yield pleasure. Indeed, we propose that one of the proximal mechanisms sustaining exploratory vocalization, occurring at massive rates in human infants and presumably continuing as solitary muttering in adults, is that, at least in part, it is fun, with endorphins being main ingredients of the sensations.

## From emotional signal to emotional language

### Differentiating features of language from features of nonhuman animal vocalization

A key difference between NHP vocal communication and an act of language is that a NHP vocal signal is coupled to a specific function or class of functions for which it was evolved. We know of no report of a NHP using a specific aggressive signal (e.g., a growl) in any non-aggressive state, except in play, where many behaviors can be used to “pretend.” Similarly, courtship signals require a state of Lust, and a call for help appears to require a state of Fear.

Language, on the other hand, in its mature form, consists of conventional and learned symbols, and these symbols can signify entities (objects, actions, states, and so on) abstractly, with no necessary connection to a social function. A language act or “Illocution” (Austin, 1962) is motivated by emotions, of course, but we can use any symbol or symbol sequence of language to express any emotional state and thus also a seemingly unlimited variety of illocutions. For example, we can use the word “mouse” in various emotional states, and thus we can transmit various illocutionary forces with the word. Someone might, based on Fear, jump onto a chair and exclaim in an illocution of alarm, “a mouse!.” A child opening a birthday present, being delighted to find a live mouse, might express an illocution of celebration, saying excitedly “a mouse!” A teacher pointing to a chart displaying rodents might produce a labeling illocution, saying “a mouse.” “Little mouse” (Mäuschen) is used in Austria as a popular illocution of endearment spoken to both children and lovers. We have used “mouse” in this paragraph as an example, with the motivation to exemplify/share information about the illocutionary flexibility of words in languages. Importantly, the word “mouse” does not change its meaning (the class of animals it makes reference to) in these different illocutionary implementations and in the different emotional states that might have motivated them; the word always refers to a special kind of rodent that we are all familiar with and that we agree implicitly to call “mouse” in the English language.

This distinction between illocutionary function and meaning (or semantics) is critical to the understanding of a fundamental difference between language and the natural communication systems of NHP and other nonhuman animals. Wild NHP transmit illocutionary forces when they vocalize or gesture, but there has been no demonstration that they transmit semantic content. Humans can communicate in some cases with illocution alone, especially with their non-verbal repertoire (grunts, screams, cries, squeals, moans, and so on), and illocutions provide the only form of communication in the human infant. But at later stages of development, linguistic utterances appear, and these utterances include semantic content, in the form of words, generally referring to entities that need not be present. Yet every use of a word can bear any one of a large number of illocutionary forces. As in the example above, “mouse” always refers to a class of animals, but in any individual speech act, the word can constitute an alarm, an insult, a threat, an endearment, an act of labeling, or merely a pronunciation of the word.

In the first year of protophone usage in infancy, humans produce a narrow range of possible illocutions, limited to expression of just a few emotional/affective states, for example anger, distress, delight, fear, comfort, and sound exploration (vocal play) (Papaeliou et al., 2002; Scheiner et al., 2006; Oller et al., 2016). Similarly, we see no reason to believe that the range of possible illocutionary forces in NHP having grown up without human training in communication is much different from the human infant range. The list of possible illocutions appears to be confined more or less to expressions of anger (threat), fear, distress, delight (celebration), contact, submission, and perhaps a very few more (Griebel and Oller, 2008). The list should be expanded to include expressions of mixed emotions/illocutions. Saliently absent in the list, however, is vocal exploration, which to our knowledge has never been reported to occur in any of our ape relatives.

The human list of possible illocutions expands vastly as language expands through syntactic constructions that combine words into sentences. Thus we become able to express illocutions that are impossible even to imagine in nonhuman animal communication systems. These illocutions include labeling, requesting, thanking, welcoming, description, criticism, praise, denial, affirmation, argument, explanation, stipulation, and many more. Illocutionary types tend to be restricted to expressing particular emotions in nonhuman animal communication (for example, threat goes with Rage, alarm goes with Fear), but in humans there are illocutions that can be motivated by any emotion. Consider an explanation. A person can give an explanation: (a) out of mere interest in exploring an idea with someone (Seeking); (b) to counter an insulting accusation made by someone (Rage); (c) to prevent someone from striking out based on a misunderstanding (Fear); (d) to provide a rational basis that someone might like to engage in courtship behavior (Lust); (e) to provide a basis for forming a friendship (Care); (f) to justify a playful wrestling activity (Play); or (g) to evaluate irrational fears or feelings of isolation (Panic/Grief). In this way the connection of vocalization to the Seeking System in humans makes it possible not only to form a vast number of signal types, but also to use those signal types with a seemingly unrestricted variety of social intentions (illocutions), motivated by any emotional or affective state.

It may be important to emphasize that NHP appear also to transmit some illocutions on the basis of different emotions on different occasions. In gesture, for example, an invitation can be made to play or to groom (Fröhlich et al., 2016; Heesen et al., 2021). In terms of Panksepp’s (1998) scheme, it would seem that a play invitation would be motivated by the Play emotion, and a grooming invitation by the Care emotion.

Humans can, by recombination of illocutionary types expressed in complex syntax, produce a large number of illocutions in a single sentence. For example, a mature language user can form a sentence constituting a request for an affirmation of an explanation of a request. Because we can, in this way, embed various illocutions in complex sentences, there is no obvious limit to how many complex illocutions are possible in human language.

The (theoretically) indefinite size of the repertoire of human illocutions reflects the similar indefinite size of the class of possible sentences that can be composed over any human vocabulary by recombination and structural embedding of phrases consisting of words (Chomsky, 1965). In fact, without complex syntax, expression of complex illocutions is not possible. And the words of which sentences are composed are themselves sequences of syllables that can be recombined to form an indefinitely large vocabulary of words (Pinker, 1994).

The recombination of discrete units at all these levels (illocution, syntax, vocabulary) is based on a digital rather than analog system that has been related to the “particulate principle” in the organization of both inorganic and organic systems. Importantly, the particulate principle in human language (Studdert-Kennedy, 2000, 2005), operating according to our reasoning on the basis of the Seeking System, makes the repertoires of language at every level vastly different from the comparatively tiny set of vocal communication types available to NHP. A key difference is that NHP vocal types seem to be obligatorily analog, offering gradations along a few dimensions of illocution but with no particulate digitization necessary for recombination that would allow indefinitely large sets of communication units.

**Figure 1:**
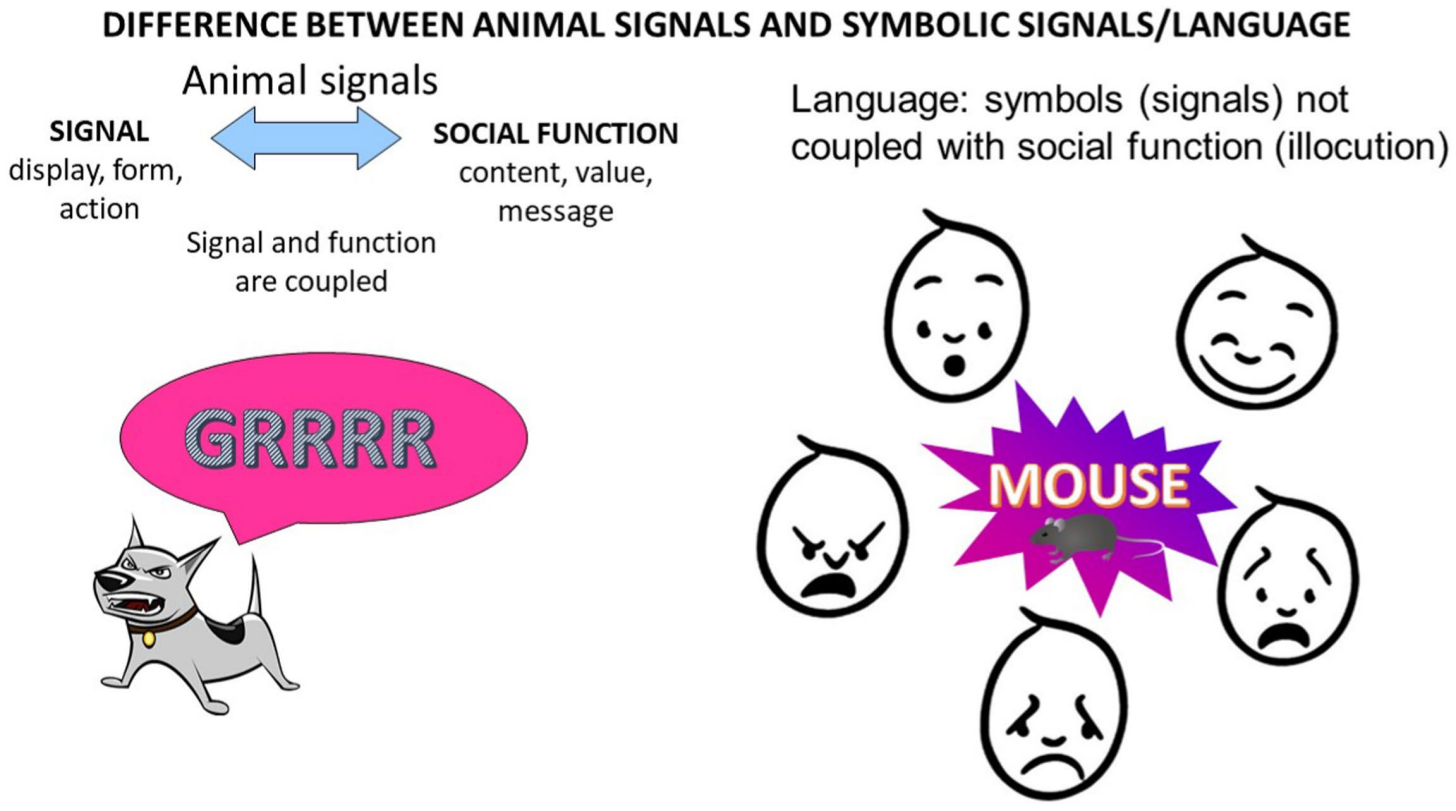
In vocal communication of nonhuman animals, each signal type (“call”) tends to be coupled with a social function or class of functions, motivated by emotion (for example, growling motivated by Rage in dogs). In language, the signal (“mouse”) and the illocutionary function, for example, expression of joy, play, fear, anger, or desire to share information, are not coupled. Linguistic signals can be used flexibly for any social function, that is, in the expression of any conceivable illocutionary force. And most importantly they can be produced purely as exploration, devoid of any social motive, proving their status of being decoupled from any immediate need and thus available for expression in any state and with any illocutionary purpose.

Figure 1 portrays perhaps the most fundamental difference between human and NHP vocal communication and corresponds to the first item on the list above of 4 ordered steps in human development. NHP signals tend to present couplings between individual action types (signals themselves) and individual social functions (or illocutions) motivated by particular emotions (or complex illocutions motivated by mixed emotions). Recent research suggests there is some, but quite limited, flexibility in these couplings in bonobos and chimpanzees (Clay et al., 2015; Taylor et al., 2023). In the human case, on the other hand, the flexibility is extreme. Every language signal type and even each type of protophone precursor to human language has no necessary coupling with any particular illocution or with any particular emotion. Because of the extremely flexible control of the vocal apparatus in humans as manifest in exploratory vocalization, humans can create new categories of sound not provided in their innate repertoire (e.g., cry or laughter). These new sounds can be graded, but they also develop into discrete syllable types by the second half year of life in canonical babbling (Oller, 1980; Stark, 1981). These discrete syllable types form the foundation for unlimited recombinations of syllables and thus the basis for an indefinitely large vocabulary. No NHP has ever been shown to develop such discrete, recombinable syllable types, although many songbirds seem to produce recombinable syllable-like elements.

Considerable research has been devoted to demonstrating that voluntary vocalization may be possible in NHP as manifest in vocal learning of, for example, either a very small number of sounds after extensive training/experience, or modifications in usage of innately available sounds after similarly long periods of experience (Hopkins and Savage-Rumbaugh, 1986; Fischer, 2003; Hopkins et al., 2007; Taglialatela et al., 2012; Hage et al., 2016). But such research has not, to our knowledge, even attempted to quantitatively compare vocal learning in NHP with that which occurs even in a young human child, who can learn a large array of new sounds or sound sequences on single trials. The very weak vocal production learning in NHP contrasts sharply with the considerable vocal learning ability of humans and with the ability of NHP to learn how to interpret sounds they hear (Seyfarth and Cheney, 2010) or to produce non-vocal (non-phonatory) sounds imitatively (Hopkins et al., 2007).

A second difference, also presented in the list of four, is that humans interact vocally with positive affect, often with eye contact. Of course eye contact does occur communicatively in NHP (Bard et al., 2005; Kano et al., 2015; Heesen et al., 2021) although it appears usually to be brief and sometimes to be avoided (Kaplan and Rogers, 2002), perhaps as threatening. In contrast, a combination of directed gaze and non-aggressive vocalization exchange in human infancy is widely recognized as beginning in the first months of life and appears to produce bonding and a growing understanding in the infant of the possibility of sharing affect and recognizing the emotions and minds of others (Trevarthen, 1974, 1979; Terrace et al., 2022). This sort of sustained, affectively positive vocal exchange has never to our knowledge been observed in apes other than humans, although it may occur to some extent in singing birds (Fehér et al., 2017; Rivera-Cáceres et al., 2018) and in the New World callitrichid monkeys (Takahashi et al., 2013, 2015).

The third item on the list of four is vocal imitation of new sounds or sound sequences not previously occurring in the repertoire of the imitator. Humans learn to imitate particular syllable sequences that later become the most primitive words. No NHP has ever succeeded in producing more than an extremely limited variety of syllables, and that limited repertoire is uncertain (Kellogg and Kellogg, 1933; Gardner and Gardner, 1969; Gardner and Gardner, 1985). In general it appears NHP learn scarcely, if at all, to produce phonatory sounds not in their innate repertoires. Among the best examples is that Hopkins et al. (2007) claimed captive chimpanzees learned an “extended grunt” (with grunts already being in the innate repertoire), which the chimps used in attention getting with human caretakers. While interesting, this is hardly a demonstration of extensive phonatory learning. In contrast, many birds, e.g., blackbirds, corvids, and parrots have learned to imitate very large numbers of human syllables and more importantly very large repertoires of other sounds not in their innate repertoires.

Human infants by the second year learn to produce words that can be decoupled from any particular social function—each word production can occur with any one of a wide variety of functions. These words constitute semantic entities, that is, vocal symbols. No NHP has ever been shown to produce words in this way, although sign language learning and other visual domain learning in nonhuman apes has clearly produced cases of some non-vocal acquisition of word-like symbolic units with at least some functional flexibility (Fouts, 1987, 1991; Griebel et al., 2016). Many birds can imitate an extreme variety of sounds (Tchernichovski et al., 2001; Dalziell and Magrath, 2012; Lipkind et al., 2013). It appears the gray parrot Alex and his parrot colleagues could be the only *animals* ever to have been shown to produce imitative vocal words with illocutionary flexibility; still, even with Alex, the number of learned words was extremely small compared with word repertoires of even three-year-old humans (Pepperberg, 2010).

Because the list of four presents sequential steps of development in humans with each subsequent step being apparently dependent on the previous ones, it should be no surprise that apes other than humans have never reached a real word vocal symbolism stage, that is, step #4. Since they have never been shown to pass the first step, there is no logical basis to expect that they could pass the more advanced steps that are also required for language. Even though great apes have been shown to possess the cognitive requirements to learn gestural symbols when taught by humans, they have not independently evolved sign language either.

But why did humans, in some distant past, ever achieve the first step? This “ultimate” question (Tinbergen, 1963; Mayr, 1993) about the evolutionary conditions required for language foundations has rarely been considered explicitly. We have proposed there is solid logical reason to believe ancient hominins could not have gone further toward language without that first step (Oller et al., 2016). What evolutionary scenario could plausibly have presented circumstances where natural selection could have driven ancient hominins to produce sounds that were decoupled from any particular emotional state or social function? And why have animals such as song birds never gone on to language, even though many of them seem to have achieved the first step, and in some cases even step #2 or #3? Some bird species are able to imitate many sounds in their environment, whether they are made by other bird species or by human contraptions. Yet even in such cases, language has not emerged. One likely reason is a lower degree of sociality in birds than in humans, but there are other possibilities such as lesser brain complexity or size, the shorter time available for development, and the number of generations required for the evolutionary steps to occur.

### Transition from emotional signal to symbol: the Fitness Signaling Theory

How was it possible for hominins to go from functionally fixed signals to functionally completely decoupled signals? Our answer has already been partially revealed, and we will suggest it also supplies a hint about how singing birds, who produce subsong (babble) prior to maturity (Ter Haar et al., 2021), may also have evolved to produce decoupled signals.

According to our proposal, the mechanisms of vocal control in ancient hominins had to be connected through natural selection to the Seeking System. We presume that the result was a much increased ability as well as an *inclination* on the part of ancient hominins to produce vocalizations that were decoupled from particular illocutionary functions and were often produced just for the pleasure of vocalizing. The decoupling presumably occurred first in infants, and those infants would have carried the decoupling capability into adulthood. Sounds thus produced through the Seeking System would have been possible to produce as endogenous exploration and in a wide variety of emotional/affective conditions both in infancy and later in life.

Why was this connection of the Seeking System to vocal production evolved in ancient hominins but not in other primates? We, along with Locke (2006, 2009) independently, have proposed that when ancient hominins became bipedal, and the hominin pelvis was necessarily changed (Washburn, 1960; Wells et al., 2012; Haeusler et al., 2021), selection pressure caused adaptations of development in the hominin infant in order to accommodate passage through the pelvis at birth (Bogin and Smith, 1996; Locke and Bogin, 2006). The combination of requirements is believed to have resulted in higher death rates in ancient hominin mothers, due to an “obstetrical dilemma” at the point of birth, and the problem persists; higher death rates clearly occur in modern human mothers at birth (Haeusler et al., 2021). More importantly for our reasoning, the accommodation is posited to have required a slowing of fetal development, resulting in a premature, altricial (helpless) newborn (Locke and Bogin, 2006; Hrdy, 2009). Furthermore, the slowing of development also yielded a longer period of relative helplessness, meaning hominin infants required (and modern human infants require) provisioning and protection for longer than their nonhuman ape relatives. That longer period was accompanied, in our reasoning, by heightened selection pressure on fitness signaling to elicit long-term investment from caregivers (Oller and Griebel, 2005, 2008; Locke, 2006, 2009).

Hominin infants thus competed against each other for care by vocalizing, since their helplessness greatly reduced their means of demonstrating wellness with physical movement (Oller and Griebel, 2014, 2021). Thus, we contend, the pressure to make vocalization more conspicuous and an increasingly more potent indicator of wellness resulted in increasing connection between the Seeking System and the vocal inclination of hominins. In this way our ancient ancestors came to use vocalization for the pleasure of Seeking and as a fitness signal, first in infancy as an advertisement that yielded caregiving, and later at all stages of maturation as an advertisement that yielded investment in the signaler in the form of mating, alliance formation, and cooperative activity. All this happened, according to our hypothesis, before language existed in the form of words and recombination of words in sentences.

This Fitness Signaling Theory (FST) is the only attempt we know of to explain the ultimate origin of exploratory vocalization in humans. The notion that exploratory vocalization could have been evolved directly *as a prerequisite for language* makes no sense, because natural selection cannot see into the future (Dawkins, 1996). If the capacity for exploratory vocalization had to evolve before language, there had to be an advantage to exploratory vocalization independent of language. The advantage of securing long-term caregiver investment through fitness signaling by altricial hominin infants suggests the solution.

We also contend that the value of fitness signaling provides a primary basis for maintaining massive vocal activity in human infants in modern times. We have plans to seek empirical support for the FST in the near future through experimentation, including monitoring physiological responses of caregivers and potential caregivers, who will listen to infant protophones in a variety of circumstances and to other kinds of sounds in the same circumstances.

Are there other reasons that vocalization evolved to become so important in human communication? Consider a property that makes vocalization particularly available for selection as a modality of communication. The vocal system in NHP is not often necessary for doing things beyond communicating (Oller and Griebel, 2021). The hands, for example, are used for manipulating objects, carrying things, climbing, and so on, and consequently hand gestures are often not available as communicative vehicles. But the vocal system, aside from its occasional functions in respiration (coughing, sneezing) and digestion (burping, hiccoughing) is available to be exploited as a signaling system because it is not required for many other functions. Perhaps all mammals have vocal signals partly because the vocal modality is almost always available to be targeted by natural selection for signaling. Thus the vocal capacity was surely open to extensive developments in ancient hominins.

Another property of vocalization that affords it an advantage over other kinds of possible fitness signaling is that receivers do not have to be looking in order to notice vocal signals. Vocal signals are effective even in the dark. These facts illustrate major advantages to vocal signaling over gestural or other visual forms of communication.

In addition, there were other conditions of life among ancient hominins favoring vocal fitness signaling in the infant. One was relatively large group sizes (Dunbar, 1996), affording protection from predators and reducing the pressure on silence to keep from alerting predators. Yet another factor was the growing tendency across hominin evolution for individuals to be cooperative breeders (Snowdon, 2004; Burkart et al., 2018), that is, many group members participated in caregiving for infants. As a result, there were many potential caregivers who might notice infant vocal fitness signals and invest more heavily in infants who produced especially effective signals.

Finally, we contend that there was an adaptation in hominins for conscious control of the glottis, owing to the same factor in hominin living that may have led to bipedalism, namely significant periods of waterside living (Hardy, 1960; Morgan, 1997; Tobias, 2002; Wrangham et al., 2009; Verhaegen et al., 2011; Joordens et al., 2019). Foraging for food by wading in water would have placed pressure on upright posture and bipedalism (Kuliukas et al., 2009), which is the apparent source of our altriciality at birth, and for the ability to consciously close off the glottis for foraging underwater in diving. The ability to consciously control glottal closure must have been accompanied by higher ability to control adduction of the vocal folds along with enhanced control of subglottal pressure required for phonation. Thus we reason that ancient hominins may have been more sensitive than other primates to natural selection pressures on fitness signaling by vocalization at the same point at which obligate bipedalism was making them more altricial.

The suggestion that diving may be not only associated with heightened conscious control of the glottis but with vocal learning in general seems plausible since many diving marine mammals are known to be vocal learners (Schusterman, 2008; Vernes et al., 2021). Of potentially similar interest is the fact that flight may also be associated with heightened conscious control of respiration and consequently with vocal learning capacities in thousands of bird species (Berg et al., 2019).

The Fitness Signaling Theory as a basis for vocal learning in humans is consistent with vocal learning in many species. The largest vocal repertoires, along with greatest variability and sources of vocal novelty are found regularly to be used for fitness advertising. Approximately 4,000 species of songbirds are believed to use their songs as signals of fitness to potential mates and as signals of their capability to protect their territories against invaders (Hausberger and Snowdon, 1997). So there is nothing that should be viewed as unusual in our proposing that human vocalization involves fitness signaling as perhaps its most fundamental motivation (Miller, 1996, 2000).

### Additional features of vocal fitness signaling

Many types of vocalizations, not merely those motivated by the Seeking System, can serve as fitness signals. For example, mating songs produced by male birds are motivated by the Lust system as portrayed by Panksepp (1998), and are selected for by females for precision, variability, creativity, and perhaps for beauty to the ear of the beholder. Thus the form of courtship song is shaped by sensory biases of female birds. Birdsong is perhaps the most widely recognized type of vocal fitness signaling in the animal kingdom (Nottebohm, 1981; Baptista and Petrinovich, 1986; Hausberger and Snowdon, 1997; Kroodsma, 1999; Catchpole and Slater, 2003). There is good reason to think of much of birdsong and subsong in fledglings as being playful, and presumably motivated by the Seeking System. It seems clear that the Seeking System is involved in cases where males need to impress their audience with novel sound types (as in the case of humpback whales) and where males need to find new and exciting sounds in their environments to imitate so they can exceed the repertoires of their rivals. The Australian lyre bird can copy anything from a chain saw to a camera shutter sound with impressive accuracy (Dalziell and Magrath, 2012).

Territorial songs and choruses may be motivated by aggression and are demonstrations of vigor, stamina, and endurance to impress either single opponents in neighboring territories or to give competing groups living in nearby territories reason to stay away. In group-living social species, territorial songs may also reinforce group cohesion. Even sounds motivated by Rage can function as fitness signals in addition to their function in intimidating the individuals targeted by the aggressive act.

Securing caregiver investment may also be a general basis for selection of fitness signaling capabilities. Many animal infants (including most birds) face competition with siblings for caregiver investment through food and protection. This is not only true for siblings in the current litter/brood/clutch but also for consecutive single offspring births over the lifespan of the caregivers. Animals invest more in healthy offspring that in sickly ones, which they often abandon. Human infant mortality is still near 50% in some places in the world, where it is known that sickly infants are often abandoned. Even in the more prosperous modern world, neglect and abandonment are still focused on infants who fail to thrive (Locke, 2011).

Infant fitness advertisement has scarcely been investigated in other species, for example in birds, where song in adulthood is critically important, and where it has been reported on the basis of a broad survey that birds who sing in adulthood have a sort of bird “babbling” (subsong) in the fledgling stage (Ter Haar et al., 2021). Could subsong have been naturally selected long ago as fitness signaling before mature bird song existed? We know that the offspring of many animals beg for food vocally, and this has been attributed mostly to hunger, but we suspect that they may also be advertising their fitness (Rodríguez-Gironés et al., 1996). This question needs to be investigated.

Vocal creativity and high volubility might be useful for the caregivers of other species to determine offspring viability. Interestingly, the only other primates known to show “babbling” in the young are apparently the New World callitrichids (Elowson et al., 1998; Ghazanfar, 2013), including the marmosets and tamarins, which share the social system of cooperative breeding with humans. Perhaps cooperative breeding encourages fitness signaling in infant callitrichids. Nevertheless, these infants do not appear to use *novel* sounds in their “babbling.” Rather they appear to use the sounds of the adult (inert) repertoire.

Human infants produce protophones from the first day of life and, if they are born prematurely, as soon as they can breathe independently (Oller et al., 2019). Protophones occur voluminously, much more frequently than any other vocalization type, thousands daily. But only a small proportion occur in interaction with caregivers (Long et al., 2020, 2022), revealing that protophones are predominantly motivated endogenously, presumably as seeking behaviors that result in parental investment even though infant protophones do not usually seem to be consciously noticed by parents. The rate of protophone production seems undiminished even when infants are alone in a room or no one is attending to them vocally (Delack, 1976; Iyer et al., 2016). According to our hypothesis, ancient hominin caregivers supplied the selection force on vocal fitness signaling in infants, but infants were capable of transmitting fitness information even when not directing the vast majority of their vocalizations to any caregiver. Ancient hominin infants broadcasted their fitness signals to anyone who *might be listening*. The same pattern of fitness signaling appears to be operative in the present, and modern human caregivers clearly show interest in the sounds their infants produce, trying to elicit them in face-to-face interaction and imitating infant sounds they have come to recognize (Stern, 1974; Trevarthen, 1979; Bornstein et al., 1992; Gratier and Devouche, 2011; Bornstein et al., 2015; Gratier et al., 2015).

Another line of reasoning that seems compatible with our proposal has been advanced by Levinson and colleagues, who have written of “cuteness selection” (Levinson, 2006a,b, 2022). They propose that human infants and ancient hominin infants may have used both protophones and other features of infancy to tap into caregiver tendencies to select infants based on emotional reactions to their lovability, which may have been, according to their reasoning, subject to runaway selection (Fisher, 1915). The idea of runaway selection incorporates integrally the notion that fitness signaling requires a real association between the signal that comes under selection pressure and wellness.

In many species, especially many species of birds, fitness signaling is seen to involve a learned and highly variable sound repertoire designed to impress potential sexual partners. It seems plausible that a learned and variable repertoire in infancy may also be used in birds, as in human infants, as fitness signals that elicit care.

### Additional steps toward language after the emergence of exploratory vocalization

The claims of the Fitness Signaling Theory differ from those of other widespread attempts to account for the evolution of language, because our proposal aims to account for a very early step, a beginning without which subsequent steps toward language would not have been possible. A great deal of publication about the evolution of language (Chomsky, 1986; Bickerton, 1990; Pinker, 1994; Harnad, 1996; Deacon, 1997; Christiansen and Kirby, 2003; Niyogi, 2006; Chater et al., 2008; Berwick and Chomsky, 2016) addresses advanced features of language such as syntax and complex vocabulary, often without even a mention of the early adaptations we have proposed in the present paper, adaptations that seem to have broken hominins away from the primate background long before there was language.

But we need a more elaborate theory of how, after the evolution of vocal fitness signaling in early hominins, the next steps toward language could have been instantiated by natural selection pressures. The necessary pressures appear not to have applied to other species with massive vocal repertoires—otherwise many birds would surely have evolved language. So there must be adaptive advantages to evolving a wide variety of additional features necessary to language (beyond exploratory, fitness signaling vocalization) that occurred in hominins but no other vocal learning species.

Consider the conventionalization of syllables and syllable sequences to create words. Many suggestions have been made about advantages of evolving words. For example, words make possible the naming of group members in order to keep track of social contacts and alliances; cooperative hunting has been thought to require words to coordinate actions; naming objects has been thought to facilitate tool use and trading objects; and there have been many other suggestions as cited in Christiansen and Kirby (2003). There have also been intriguing suggestions about how prosociality of hominins and their presumably cooperative tendencies may have supported evolution in the direction of language (Tomasello, 1996, 2008; Tomasello et al., 2005; Nowak and Highfield, 2011; Kaplan, 2014, 2023). Note that all these suggestions appear to be dependent on *highly social conditions.* Indeed, the advantages of vocabulary and later syntax are obviously increased with increasing complexity of culture.

Hominin groups appear to have increased in size across evolution (Dunbar, 1993, 1996), and consequently their cultures must have been complex, with complex communicative needs. One special need in primates is grooming, and as hominin groups increased in size, Dunbar (1993), argued they may have come under pressure to use vocalization as a substitute for grooming, since friendly social vocalization could be transmitted to multiple individuals simultaneously. The argument reinstates an earlier notion of “grooming talking” in ancient hominins (Morris, 1967). After vocal fitness signaling was evolved, and flexible vocal repertoires were available, it appears increasing needs for more powerful communicative capabilities made ancient hominins sensitive to natural selection pressures that promoted the expansion of their vocal repertoires and the building of vocabulary and syntax from them, which would have served both fitness signaling and “grooming” needs.

It seems likely that one reason birds have not evolved language is that their lifestyles never had the range of social interrelations and consequent advantages of coordinated action that occurred in ancient hominins. Interestingly, the largest vocal repertoires in nonhuman animals do not coincide with a high degree of sociality. Some of the most elaborate known mating songs occur in species where potential mating partners see each other only once a year. For example, among marine mammals, mating songs have evolved in some solitary living baleen whales (Simon et al., 2010; Stafford et al., 2018) as well as in seals (Bjørgesæter et al., 2004) and walruses (Sjare et al., 2003), but not in the extremely socially-living dolphins. The same is true for the socially living parrots, which have elaborate social vocal repertoires. But their vocalizations in mating are not generally treated as “mating songs” although they are used to coordinate mating (Spoon, 2006). We suspect that very socially living animals do not require mating songs since potential mates know each other so well they can assess fitness of individuals based on long-term experience with them (personal communication, Drew Rendall). In contrast, if animals meet their potential mates only once a year, an elaborate song and/or dance may be necessary to provide the fitness information for mate selection.

A comment is called for regarding “signature whistles,” vocalizations that appear to be indicators of the identity of individual bottle-nosed dolphins, which are highly social and strong vocal learners (Caldwell and Caldwell, 1965; Quick and Janik, 2012; Janik and Sayigh, 2013; Fearey et al., 2019). Each individual is claimed to invent a signature whistle by modification of other whistles. Signature whistles constitute around half the whistles produced by free-ranging dolphins and a much larger proportion in captive dolphins isolated from conspecifics. The whistles produced by dolphins in isolation have been interpreted as attempts to make contact with the prior group. Other members of the group are reported to use a slightly modified “copied” version of the individual’s whistle perhaps to call the individual (Janik and Sayigh, 2013). The copying has been interpreted as “reference” to the individual and thus has been taken as a limited indication of semantics in wild dolphins. It should be noted that there have been empirical challenges to the very existence of signature whistles (McCowan and Reiss, 1995, 1997), but the claim of their existence remains a suggestion of semi-semantic evolution in nonhuman animals. There is also evidence that bottlenose dolphins can learn to associate other specific whistles as well as visual symbols with specific objects through operant conditioning (Herman and Forestell, 1985), an achievement that suggests parallels to the learning of visual symbols in chimpanzees, bonobos, and gorillas; see review in Tomasello (2017). Killer whales have family group repertoires of discrete whistles that partially overlap with those of closely related groups, but not with those of strangers (Ford, 1991). In social dolphins, all group members use all the whistles of the group as well. Thus for scientists to discriminate between usage as individual signature whistles or group marking repertoires, a whistle discrimination experiment in dolphins would be useful. If it could be shown that dolphins do use certain calls as signatures, this would be indeed a limited case for “naming” in the wild.

Apes other than humans are, like dolphins, extremely social and intelligent. But they appear never to have evolved a basis for complex vocal communication because, we contend, they never evolved a large vocal fitness signaling repertoire. Perhaps because they were less altricial at birth than hominins, because their group sizes were smaller, and because they showed less cooperative breeding, there was not sufficient pressure to evolve creative vocal fitness advertising. Even more important, as far as we know, no NHP evolved an extensive adaptation for voluntary control of the glottis.

### Vocalizations integrated across a wide range of emotions

Through the connectivity of the Seeking System to vocal control, humans are motivated not only to explore sounds they essentially invent in vocal exploration, but also to explore the originally innate sounds associated with emotions such as Fear or Rage. Mature humans (especially actors) can toy with gradations between such sounds at will, can combine them in alternating patterns and use any of them in any emotional state. All of us can pretend to be crying or laughing at a chosen level of intensity (some more convincingly than others), and we can perform these vocal acrobatics even while we are talking.

Thus humans can add emotional flavoring in the form of prosodic contours or variations in pitch or amplitude when producing any kind of sound, including speech or otherwise innate signals such as shrieks, moans, or laughter. Humans also conventionalize various sounds drawn from the innate repertoire when, for example, saying “ha” (suggesting laughter) but invoking some special intended meaning, for example triumph, as in “I got you!.” Or we can growl in rough and tumble play, or vocalize with pleasure during a massage, copulation, or in the anticipation of tickling. Everyday prosody often reflects the emotional state, the motivation and often the intended illocution of the sender in speech, a pattern that in some cases results in language-specific “pitch accents” (Pierrehumbert, 1979; Pierrehumbert and Hirschberg, 1990; Gussenhoven and Jacobs, 1998).

No other mammal appears to have such vocal flexibility. No other primate has a large repertoire of discrete syllable types nor an indefinitely large repertoire of syllable sequences, decoupled from any particular function. Instead, other primates have small repertoires of vocal types, each of which is graded to serve a relatively narrow class of possible functions. Human signals can also be graded, and the possibility of gradedness applies, not just to specifically emotional signals such as crying or laughter, but to every syllable, every word, every sentence of any language, all of which are, in accord with our proposal, possible to modulate through the Seeking System.

## Conclusion

### Summary

Much misunderstanding in the attempt to understand nonhuman animal and human communication has been caused by terminological missteps. Nonhuman animal signals are overwhelmingly about emotional states and illocutions, rather than constituting symbolic/semantic elements that must be detachable from emotional states and their accompanying illocutionary forces. Linguistic terms such as “reference” or “syntax” that have often been used in describing nonhuman animal vocalization are confusing rather than clarifying. Furthermore, research that restricts interpretation of vocal behavior to external observable actions occurring in particular situational contexts represents a failure to even address the primary goals of evolutionary science. We must develop understanding of the functions and motivations underlying vocal behavior if we are ever to develop a workable theory of the evolution of communication.

We have proposed that each call type is coupled to particular emotional/motivational states in nonhuman animals. Such vocal signals must be flexible enough to express, for example, gradations of intensity, to allow for audience effects, and to allow expressions that reflect mixed emotions. Humans also possess vocal signals, such as cry and laughter, that are commonly coupled to particular functions, and these signals have very similar properties to those of NHP. But even cry and laughter become very flexible in humans beyond early infancy. How did this occur in evolution?

We hypothesize that hominin vocal communication first diverged from the vocal communication of our competitor primates through the evolution of fitness signaling in primarily exploratory vocalizations. We have proposed that this divergence required a naturally-selected connection between the Seeking System proposed by Panksepp (1998) and the vocal control system of ancient hominins, making it possible for hominins to produce a wide variety of sounds that were decoupled from any particular emotional state or illocutionary intent. This decoupling allowed hominins to evolve further flexibility of vocalization, making possible learned vocalizations that could be used in any emotional state. The beginning of the break with the primate background appears to have occurred in the altricial hominin infant, who was selected to vocalize exploratorily and plentifully, thus maximizing the likelihood of long-term investment from caregivers, who noticed the vocal expression of well-being in the infant sounds.

Hominin infant development of vocal fitness signaling constituted the *first step* in producing a flexible learned and large vocal repertoire, according to the Fitness Signaling Theory. Subsequent steps were necessary because a large vocal repertoire does not by itself yield language. Additional steps were presumably naturally selected because of advantages of complex communication in the highly social, cooperatively-breeding hominins. A wide variety of social functions, such as group protection, hunting, foraging, tool use, vocal grooming, and trading were promoted by vocal signals that were possible to create once exploratory vocalization was established deeply enough to allow the evolution of words and sentences.

### Looking forward

The Fitness Signaling Theory, largely in agreement with a similar proposal by Locke (2006), represents a departure from the predominant trend in research on primate communication, a trend that utilizes the misleading terminology critiqued in our article. The trend seems to apply blinders to its proponents by encouraging attention only to communicative similarities between humans and other animals to the practical exclusion of addressing the important differences. The approach suggests there is something unseemly about investigating human uniqueness, as if to do so would require us to go back to thinking in ways that were common two centuries ago. The current predominant trend thus becomes, in our view, hidebound, rejecting one of the most fundamental goals of biological research, which is to account, whenever possible, for species differences in terms of adaptation. Language is a massive adaptation, treated by some biological theorists as one of the major transitions since the origin of life (Maynard Smith and Szathmáry, 1997; Maynard Smith and Szathmáry, 2000; Szathmáry, 2015), and we are trying to account for the most fundamental adaptive changes that laid groundwork for the evolution of the whole range of language capabilities.

Consider an analogy: ancestral saurischians (reptile-hipped dinosaurs) did not fly, yet the only surviving descendants of dinosaurs are believed to be thousands of species of birds (Padian and Chiappe, 1998). Flying in vertebrates is a major adaptive change, worthy of major scientific attention. The current account suggests feathers were first evolved for functions such as thermoregulation. The reasoning that goes into this account invokes symmetry of feathers in the first saurischians that had them and descent by modification to yield additional adaptive steps toward feathers with asymmetrical features compatible with flight (Prum, 1999; Benton et al., 2019). We suggest language deserves a similar scientific effort, and the Fitness Signaling Theory represents a proposal for how adaptations necessary for language were first selected.

It is as if advocates of the predominant trend of research on primate communication deny that language is a major adaptation, because they seek to show that nonhuman primates possess all the fundamental features of language. Thus the advocates deny the importance of developing a strategy that might lead to evolutionary explanation as has occurred in evolutionary research on avian flight. The denial is not explicit but is instead implemented in a research strategy where attention is not focused on the nature of language as an adaptation that goes vastly beyond the communicative capabilities of NHP or any other animal.

A workable account of the human language adaptation requires recognition and detailed portrayal of the nature of the differences as well as the similarities between language and vocal communication in our closest relatives. We see hopeful signs in research on primate communication, because a few recent articles (Clay et al., 2015; Dezecache et al., 2020; Taylor et al., 2022, 2023) have begun to address the possibility of vocal functional (or illocutionary) flexibility in our ape relatives. We hope that beginning will soon lead to a more concerted effort to develop a truly comparative enterprise where the origins of language are assessed in a broader evolutionary perspective and through direct empirical studies of vocal communication of both our ape relatives and ourselves.

## Data availability statement

The original contributions presented in the study are included in the article/supplementary material. Further inquiries can be directed to the corresponding author.

## Author contributions

UG and DO wrote the manuscript and conceived of the theoretical framework it expresses. All authors contributed to the article and approved the submitted version.
